# The validity of the renal Doppler resistivity index in renal allograft infections

**DOI:** 10.1080/0886022X.2025.2462443

**Published:** 2025-02-06

**Authors:** Moataz Fatthy, Karim M. Soliman, Éva Csongrádi, Abd El Rahman Marzouk, Ahmed Fathy, Ahmed Fayed

**Affiliations:** ^a^ Nephrology Unit, Internal Medicine Department, Kasr Alainy School of Medicine, Cairo University, Cairo, Egypt; ^b^ Division of Nephrology, Department of Medicine, Medical University of South Carolina, Charleston, South Carolina, USA; ^c^ Medical Services, Ralph H. Johnson VA Medical Center, Charleston, South Carolina, USA; ^d^ Centre for Translational Medicine, Semmelweis University, Budapest, Hungary; ^e^ Institute of Pancreatic Diseases, Semmelweis University, Budapest, Hungary

**Keywords:** Renal resistive index, renal allograft infection, CMV, BK virus

## Abstract

**Background:**

The correlations between the intra-renal resistive index (RRI) and renal histopathology characteristics, especially in those with infected allografts, have not been sufficiently investigated in renal transplant recipients. We aimed to examine the correlation between RRI and renal allograft infection in these subjects.

**Methods:**

One hundred nine renal allograft recipients were recruited, and RRI was evaluated for correlation with renal allograft infection. Based on laboratory and histopathological findings, 64 renal-allograft recipients were recruited for the infected group, and 45 were recruited for the non-infected group.

**Results:**

The causes of allograft infection were Cytomegalovirus (CMV) infection (30.3%), urinary tract infections (UTI) (18.3%), and polyomavirus 1 (BK virus) infections (10.1%). There was a statistically significant difference in RRI in those with allograft infections, with the ROC curve for detection of infection utilizing RRI demonstrated an Area Under Curve 0.634 (*p*-value 0.015; cutoff value: 0.765; CI:0.527–0.742), with a specificity of 64.4% and a sensitivity of 68.8%.

**Conclusion:**

Normal renal graft arterial resistivity index values, despite a renal allograft dysfunction, may be indicative of allograft infection, guiding clinicians’ decisions regarding kidney biopsy and facilitating further biopsy interpretations.

## Introduction

Doppler imaging and ultrasound are noninvasive procedures that have long been utilized to assess chronic kidney disease. Doppler ultrasonography detects not only changes in blood flow at the macrovascular level, but also renal microscopic vascular abnormalities, with an evaluation of the vascular impedance in various renal parenchymal regions, including at the level of arcuate or inter-lobar arteries in the upper, mid, and lower kidney poles [1].

One key application of the Doppler ultrasound is the ability to calculate the RRI, a potential indicator of intra-renal parenchymal conditions. Customarily, RRI is calculated by the ratio of the difference between peak systolic and end-diastolic velocities to peak systolic velocities. Nephrologists typically do not consider an RRI lower than 0.7 as an indication of significant kidney impairment.

Some studies have found a link between high RRI and the development of glomerular disease, glomerulosclerosis, arteriolosclerosis, and interstitial fibrosis, whereas others have not [2–4]. Doppler ultrasonography measurement of the intra-renal resistive index is a common imaging technique to assess acutely impaired kidney function, including in cases of acute kidney injury and critical illness and after renal transplantation [5–7].

Subnormal values of the intra-renal resistivity index (lower than 0.5) raise the possibility of graft artery stenosis [8]. Given that the resistive index is influenced by vascular compliance, some have proposed renaming it the ‘impedance index’ to better reflect its relationship to vascular function [9]. Moreover, inflammation within the kidney is thought to affect vascular tone and endothelial function, with chemokines and inflammatory mediators playing key roles in this process [10].

In our paper, we aimed to investigate the correlation between RRI values and the presence of renal allograft infection, hypothesizing that altered RRI values may serve as an early indicator of infection in renal transplant recipients.

## Materials and methods

Between October 2021 and July 2023, this study was carried out at Cairo University’s Internal Medicine Departments. The Kasr Alainy School of Medicine Ethics Committee of Cairo University in Egypt revised and approved the study protocol under ethical approval number MD-227-2021. The necessary sample size was calculated to be 40 individuals in each group to reach a 90% power with a two-sided significance level of 5% and a standardized effect size of 0.5. We included renal transplant patients with acute allograft dysfunction and we excluded recipients with chronic allograft rejection.

Out of 109 renal allograft recipients who presented to the Department of Nephrology at Cairo University Hospital, 64 were assigned to the infected group based on laboratory and histopathological findings, while 45 were assigned to the non-infected group. All patients in this study received a living donor renal transplant.

All participants were assessed with a detailed medical history, including a review of transplantation history, duration of renal transplantation, induction therapy, immunosuppressive drugs, or history suspected of allograft infection, and received a comprehensive physical examination to detect any source of infection, as part of their routine admission care. Laboratory investigations were performed to measure serum creatinine, total leukocyte count (TLC), C-reactive protein (CRP), serum procalcitonin, urine analysis and urine culture, drug levels for tacrolimus (FK506, Prograf) or cyclosporine trough (C0) levels, CMV PCR, BK virus PCR, HBsAg, HCV Abs, HIV Abs and PCR for SARS‑CoV‑2 virus. Renal allograft biopsy results were interpreted by expert nephro-pathologists as part of the routine post-biopsy specimen assessment. Three measurements of RRI were calculated using Color Doppler Sonography (CDS) for each renal graft, and the mean value was adopted for the record. RRI was calculated using the following equation: RRI = (peak systolic velocity-end diastolic velocity)/peak systolic velocity.

### Statistical analysis

The statistical software for the social sciences (SPSS) version 28 (IBM Corp., Armonk, NY, USA) was used to code and enter the data. The mean and standard deviation, the unpaired t-test, the non-parametric Mann-Whitney test, and the Chi-square (x2) test were used for interpreting the collected data. To determine the appropriate cutoff value of RI for the detection of graft infection, an ROC curve was created and an AUC analysis was also conducted. Statistical significance was defined as a p-value lower than 0.05.

## Results

Table 1 provides a summary of the demographic and baseline laboratory information of the studied patients, the mean age of the infected group being 32.25 ± 8.41 years and 32.71 ± 7.58 years (*p*-value: 0.770) in the non-infected group, the duration of kidney transplantation of the infected group being 4.91 ± 1.70 years and 4.71 ± 1.46 years in the non-infected group (*p*-value: 0.517), CRP for the infected group was 98.22 ± 69.85 mg/dl and 4.22 ± 1.88 mg/dl in the non-infected group (*p*-value< 0.001). 64.1% of the infected group was male and 35.9% female, while the non-infected group consisted of 60% men and 40% women (*p*-value: 0.666). The causes of allograft infection were Cytomegalovirus (CMV) (51.6%), urinary tract infection (UTI) (18.3%), and polyomavirus 1 (BK virus) (17.2%). There is a statistically significant difference regarding the renal resistive index (*p*: 0.030) between the infected and the non-infected group, which is parallel to the statistically significant difference regarding CRP and pro-calcitonin (*p* < 0.001 for both) between the infected and non-infected groups. The tests for HCVAb, HBsAg, HIV, and SARS‑CoV‑2 virus PCR were negative in all studied renal-allograft recipients.

**Table 1.** Demographic and baseline laboratory data of the studied patients.

Variables	Infected group (*n* = 64)	Noninfected Group (*n* = 45)	*P*-value
Age (Years) (Mean ± SD)	32.25 ± 8.41	32.71 ± 7.58	0.770
Duration of transplantation (Years) (Mean ± SD)	4.91 ± 1.70	4.71 ± 1.46	0.517
Male (Number (%))	41 (64.1)	27 (60)	0.666
Female (Number (%))	23 (35.9)	18 (40)	
Creatinine (mg/dL) (Mean ± SD)	3.88 ± 1.13	3.88 ± 1	0.984
TLC (×10^9^/L) (Mean ± SD)	8.72 ± 4.78	7.10 ± 1.29	0.012
CRP (mg/dL) (Mean ± SD)	98.22 ± 69.85	4.22 ± 1.88	< 0.001
Serum procalcitonin (ng/mL) (Mean ± SD)	0.95 ± 1.66	0.02 ± 0.01	< 0.001
FK506 (ng/mL) (Mean ± SD)	6.92 ± 1.49	5.85 ± 1.17	0.001
C0 level (ng/L) (Mean ± SD)	90.70 ± 15.3	87.38 ± 5.68	0.359
CMV PCR positive (Number (%))	33 (51.6)	0 (0)	< 0.001
BK virus PCR positive (Number (%))	11 (17.2)	0 (0)	0.002
UTI (Number (%))	20 (31.3)	0 (0)	< 0.001
RRI (Mean ± SD)	0.74 ± 0.09	0.78 ± 0.08	0.030

TLC = Total leukocyte count; CRP = C-reactive protein; FK506 level = tacrolimus level; C0 = cyclosporine trough; CMV = Cytomegalovirus; BK virus = polyomavirus 1; PCR = polymerase chain reaction; hemoglobin; UTI = urinary tract infection; RRI = Renal Resistive Index; SD = Standard Deviation.

Table 2 presents a summary of the pathological findings from the renal allograft biopsy results. A highly significant difference (*p* < 0.001) was observed between the infected and non-infected renal allograft groups regarding the biopsy results for acute antibody-mediated rejection, acute interstitial nephritis, BK virus nephropathy, chronic antibody-mediated rejection, CMV nephropathy, and mixed rejection.

**Table 2.** Renal allograft biopsy results.

Variables	Infected group (*n* = 64)	Noninfected group (*n* = 45)	*p*-Value
Active ABMR (Number (%))	0 (0)	7 (15.6)	<0.001
Acute interstitial nephritis with neutrophils, bacterial infection (Number (%))	20 (31.3)	0 (0)	
Acute TCMR (Number (%))	0 (0)	7 (15.6)	
BK-virus nephropathy, SV40 positive (Number (%))	11 (17.2)	0 (0)	
Chronic ABMR (Number (%))	0 (0)	19 (42.2)	
Chronic active ABMR (Number (%))	0 (0)	8 (17.8)	
CMV nephropathy (Number (%))	15 (23.4)	0 (0)	
Mixed rejection (Number (%))	0 (0)	4 (8.9)	
Tubular injury, viral infection (Number (%))	18 (28.1)	0 (0)	

ABMR = antibody-mediated rejection; TCMR = T cell-mediated rejection; BK-virus = polyomavirus 1; SV40 = simian vacuolating virus 40; CMV Cytomegalovirus.

In the infection group, acute interstitial nephritis with neutrophils and bacterial infection were the most common findings. (31.3%), while among the non-infected group, chronic antibody-mediated rejection was the most common finding (42.2%).

**Figure 1.**
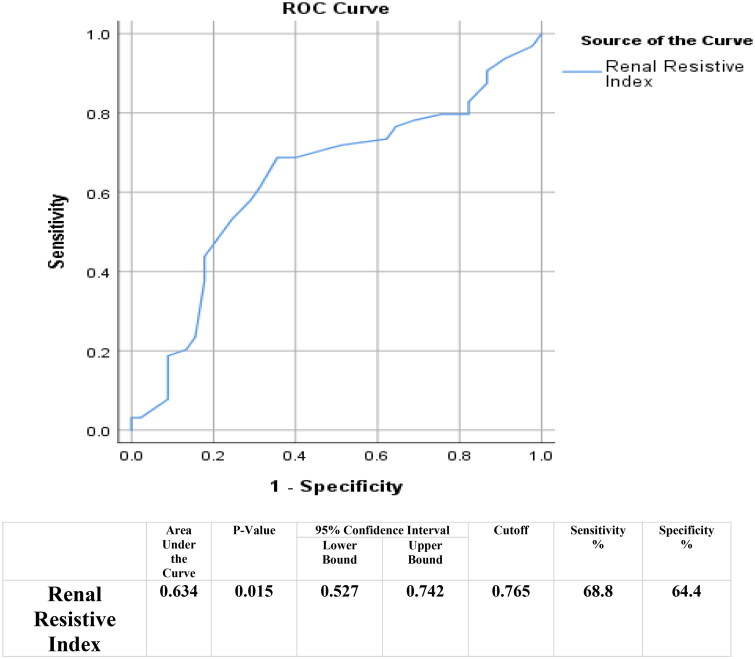
ROC curve for infection detection using the renal resistive index (RRI).

RRI was highest in the severe fibrosis group, followed by moderate fibrosis, and lowest in the mild fibrosis group, with statistically significant differences observed between the fibrosis grades. The optimal RRI cutoff value for detecting infection was determined through the area under the curve (AUC) analysis, which generated the ROC curve. The optimal cutoff value was found to be 0.765, with an AUC of 0.634, sensitivity of 68.8%, and specificity of 64.4% (Figure 1).

## Discussion

Many infections create difficulties when researchers examine rejection through histological studies. Infections can create such inflammatory reactions throughout organs that clinicians may mistake them for rejection symptoms making it difficult to establish proper diagnoses. A combined inflammatory response because of infection often confuses physicians about diagnosing rejection correctly. Microbial infections have the ability to modify immune responses which might result in switching immunological activity from increasing rejection behavior toward infection control mechanisms. Research demonstrates infections have the power to activate immune exhaustion as well as re-route immune responses [11].

As immunocompromised patients have limited tolerance to invasive infection, experience high morbidity, and show a high mortality rate, early diagnosis is essential for guiding appropriate antimicrobial therapy. Viral infections, in particular, may contribute significantly to graft dysfunction, rejection, systemic complications, and an increased risk for other opportunistic infections, cancers, and mortality. Doppler ultrasonography provides valuable, immediate, and repeatable insights into both structural and functional changes at a macrovascular and microvascular level within the kidneys, offering key diagnostic and prognostic information [1]. In this present study, we evaluated the correlation between the RRI and renal allograft infection, confirmed through laboratory workup and histopathological findings. Our study revealed a significant difference in RRI between infected and non-infected renal allografts.

Previous studies have explored the relationship between RRI and renal graft pathology. Kirkpantur et al. (2008) found that arteriosclerosis, glomerulosclerosis, and interstitial infiltration significantly influenced RRI in kidney transplant patients. The degree of tubulointerstitial injury, which is closely linked to RRI, serves as an important predictor of renal impairment and long-term graft outcomes. This latter study also revealed a mean RRI of 0.69 ± 0.12, with RRI values over 0.75 correlating with a higher incidence of globally sclerosed glomeruli and severe arteriolosclerosis [12]. Despite RRI’s limitations in characterizing the precise causes of graft dysfunction, it remains a valuable tool for predicting renal graft outcomes.

In contrast, Breitenseher et al. (2001) evaluated the long-term follow-up of 210 renal transplant recipients using CDS to measure RRI. They found that RRI values did not significantly differ between patients with normal (68.2% ± 7.5%) and malfunctioning grafts (68.5% ± 8.5%), highlighting the limitations of RRI in distinguishing graft function in the long term. Furthermore, the sensitivity and specificity of RRI for diagnosing renal allograft failure were found to be suboptimal (36% and 62%, respectively) [13]. Similarly, Barquin et al. (2013) noted that while RRI can be useful in identifying abnormal graft function, renal biopsy is still required for a definitive diagnosis, particularly when histological abnormalities are suspected [14].

In line with our findings, other studies have highlighted the relationship between RRI and glomerulosclerosis, arteriolosclerosis, and other renal vascular changes. Kang et al. (2010) found that RRI significantly correlated with the degree of glomerular and peritubular capillary loss in patients with worsening renal function, suggesting that RRI is a reliable indicator of renal damage. RRI has been linked to glomerulosclerosis and vascular abnormalities in several studies. RRI was found to be substantially associated with glomerulosclerosis and arteriolosclerosis [15].

Additionally, Restrepo et al. (2014) emphasized that factors such as the recipient’s age and hemodynamic status could influence Doppler ultrasonography results; nevertheless, intra-renal arterial Doppler proved to be a useful diagnostic tool for evaluating transplant kidney function [16].

In contrast to our findings, Naesens et al. (2013) suggested that the resistive index, typically evaluated at predetermined intervals following transplantation, reflects recipient factors rather than graft characteristics. The study found that RRI was not significantly related to histologic findings at protocol-specified biopsy intervals. The most significant predictor of a higher RRI was recipient age (*p* = 0.001). They also reported that antibody-mediated rejection (ABMR) and acute tubular necrosis (ATN) were associated with higher RRI values at the time of graft dysfunction diagnosis (0.87 ± 0.12 vs. 0.78 ± 0.14 [*p* = 0.05] and 0.86 ± 0.09 vs. 0.78 ± 0.14 [*p* = 0.007], respectively) [5].

A high concentration of CNI represents a key diagnostic characteristic in infection group even when results show normal or decreased RI values. Grafts infected with disease displayed normal or lowered RI values regardless of high CNI concentration levels. The vasodilatory mechanism produced by infection seems to overtake the vasoconstrictor action of CNI medications. Similarly, Jimenez et al. showed that several types of graft dysfunction, including acute rejection, calcineurin inhibitor (CNI) toxicity, severe ATN, renal vein obstruction, ureteral obstruction, and pyelonephritis, were associated with an elevated RRI (>0.9). They proposed that periodic post-transplant RRI testing could be valuable for the early detection of these complications, though they also found that RRI was not a reliable marker for chronic allograft nephropathy in protocol biopsies [17].

Subnormal RRI values (lower than 0.5) have been linked to the possibility of graft artery stenosis [8], and the ‘resistivity index’ name may need to be reconsidered, with some suggesting it to be renamed the ‘impedance index’ due to its relation to vascular compliance [9]. Additionally, inflammation driven by chemokines and inflammatory mediators can influence vascular tone and endothelial function [10].

**Figure 2.**
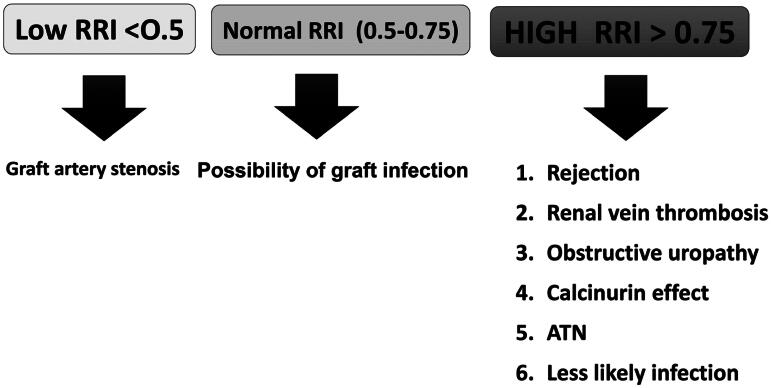
The suggested algorithm in the case of renal allograft dysfunction. RRI = Renal Resistive Index; ATN = acute tubular necrosis.

While RRI can provide useful insights, it should not be the sole factor in diagnosing graft infection. Other biomarkers, such as procalcitonin and CRP, can sometimes be misleading - procalcitonin may be negative in viral or fungal infections, and CRP is nonspecific and may rise due to another infectious or inflammatory event, not related to the well-being of the allograft. Given that transplant recipients are often immunosuppressed, the immune response to infections may not be clear. In cases of suspected infection, a normal RRI could help guide histopathologists when both infection and rejection are present in the biopsy. Our study’s findings are consistent with some of these earlier studies, but they also contributes a novel perspective on the relationship between RRI and renal allograft infection. We found that an RRI value greater than 0.765 had a sensitivity of 68.8% and a specificity of 64.4% for detecting renal allograft infection, with an AUC of 0.634 (*p* = 0.015). These results suggest that RRI could be a helpful adjunct in identifying graft infection, especially when other clinical markers are inconclusive. Our study proposes an algorithm for managing renal allograft dysfunction (Figure 2). The main limitation of our study was the small sample size, which limits the generalizability of our findings. To better understand the impact of the RRI in the diagnosis of renal allograft infection, additional studies and a larger number of patients are needed. Finally, it is important to note that RRI alone should not be applied for diagnosing graft infection, as it can be influenced by multiple factors, such as inflammation and vascular changes, which may not always be specific to infection. Further research may be needed to determine the reliability of the resistivity index in diagnosing renal allograft infection and to determine its role in clinical decision-making.

## Conclusion

Normal renal graft arterial resistivity index values, despite renal allograft dysfunction, may suggest the presence of allograft infection, helping clinicians make informed decisions about performing a kidney biopsy and aiding in the interpretation of biopsy results.

## Data Availability

This material has not been published previously, in whole or part, and is not under consideration for publication elsewhere. This paper has no tables or figures that would require permission to reprint.
